# Measurement Equivalence Across Sex, Race/Ethnicity, and Intersectional Identity for the Perceived Discrimination Scale Among Middle-School-Aged Black, Latinx, and White Youth: Findings From the Adolescent Brain Cognitive Development Study

**DOI:** 10.1007/s40615-026-02938-8

**Published:** 2026-04-13

**Authors:** Carolyn E. Sartor, Margret Z. Powell, Nicole Kennelly, Tammy Chung, Shawn J. Latendresse

**Affiliations:** 1 Institute for Health, Health Care Policy and Aging Research, Rutgers University, 112 Paterson Street, New Brunswick, NJ 08901, USA; 2 Baylor University, TXWaco, USA

**Keywords:** Racial discrimination, Measurement bias, Race/ethnicity, Gender, Youth

## Abstract

The aims of the current study were to identify and adjust for measurement non-equivalence (i.e., bias) with respect to sex (as a proxy for gender), race/ethnicity, and intersectionality (sex by race/ethnicity) in the Perceived Discrimination Scale (PDS) for middle-school-aged Black, Latinx, and White youth. Data were drawn from Follow-up 2 of the Adolescent Brain Cognitive Development Study (*n* = 9,360; mean age = 12.03, SD = 0.10; 47.53% female; 15.90% Black, 22.42% Latinx, and 61.68% White). Moderated nonlinear factor analysis was applied to the 7-item scale. Measurement non-equivalence was observed exclusively with respect to sex for two items: unfair or negative treatment because of their ethnic background (1) by teachers and (2) by other students. Measurement equivalence with respect to race/ethnicity was observed at both the factor and item levels. Adjusting PDS scores for the modest degree of measurement non-equivalence shifted the effect size from the small to medium range for differences between Latinx and White youth (0.40 to 0.52) and from the medium to large range for differences between Black and White youth (0.770 to 0.893) - a meaningful change in how these group differences would be characterized. Findings indicate that the PDS captures racial discrimination similarly across Black, Latinx, and White middle school-aged youth. Importantly, they also demonstrate the impact of modest measurement bias on the validity of comparisons across racial/ethnic groups, underscoring the importance of assessing and adjusting for measurement non-equivalence as a standard practice.

## Introduction

### Prevalence of Racial Discrimination Among Youth Across Race/Ethnicity and Sex/Gender

Racial discrimination is a social determinant of health with negative impacts on youth mental health [1–3]. An estimated 45–56% of youth of color report experiencing racial discrimination [1]. Data from the National Survey of Children’s Health, a large nationally representative study of youth in the U.S [4], indicated that racial discrimination was more commonly endorsed by Black youth relative to Latinx or Asian youth. However, another nationally representative study conducted by the Centers for Disease Control and Prevention (2021 Adolescent Behaviors and Experiences Survey) found that a higher proportion of Asian relative to Black high school students reported experiencing racism [2]. Some studies suggest that the prevalence of racial discrimination differs across gender, although again, the evidence is mixed. Notably, the presence and direction of gender differences (i.e., higher versus lower prevalence among female relative to male youth) has also been found to vary by racial/ethnic group [2, 5], underscoring the importance of considering intersectional identities when drawing comparisons across gender and race/ethnicity. Importantly, the validity of these comparisons is dependent on accurate measurement of the construct of racial discrimination across all groups under study, that is, whether the racial discrimination measure evidences measurement equivalence.

### What is Measurement Equivalence?

Measurement equivalence, also known as measurement invariance, is a psychometric property of a measurement instrument indicating that a singular construct is being measured across specified groups [6]. Measurement equivalence testing is a post-hoc statistical approach to assessing the extent to which a measure is interpreted in a conceptually similar manner by respondents with different cultural backgrounds or social identities, including race/ethnicity or gender [7]. As such, it is critical to minimizing measurement bias. Specifically, measurement equivalence methods provide a means of comparing the construct across attributes of interest, such as race/ethnicity and gender, at both the factor and item levels. That is, potential sources of bias can be assessed in relation to the average level and degree of variation in the latent construct (i.e., factor mean and variance), as well as the expected level of response and magnitude of association with the underlying construct for individual indicators (i.e., item intercepts and factor loadings, respectively).

Findings of measurement non-equivalence suggest that there is systematic bias in the measure [8], raising questions about the validity of reported group differences on the measure. Thus, we consider measurement equivalence to be best practices not only from a methodological perspective, but also from a health equity perspective. As a data-driven approach, there does not need to be a theoretical basis for the possibility that groups being compared would respond differently to the measure. Further, results do not reveal the nature of any identified bias, which is likely complex, given the multifaceted societal level processes underlying social identities such as race/ethnicity.

Identifying items that contribute to measurement non-equivalence is an essential step toward addressing measurement bias. When such items are identified, a common practice is to recommend omitting those items when administering the scale to the subpopulation for which measurement non-equivalence was observed or to refrain from administering the scale to that subpopulation altogether. Although sound advice, neither recommendation leads directly to the production of scale scores that can be validly compared across subpopulations of interest. Applying a method that involves retaining all items and original item scales was especially important in the current study, given our aim of providing a method for valid use of frequently used publicly available data with a centralized system of scale construction. We therefore selected moderated nonlinear factor analysis (MNLFA [9]), a method adopted to address the issue of deriving scores adjusted for measurement non-equivalence [10]. MNLFA can generate factor scores that account for both item- and factor-level measurement non-equivalence attributable to multiple contextual factors, that is, adjusting for systematic bias related to the social identity or identities of interest.

### The Application of Measurement Equivalence Methods to Racial Discrimination Measures

We are aware of only one study assessing the psychometric properties of the discrimination scale examined in the current investigation, the Perceived Discrimination Scale (PDS; [11]), that included assessment of measurement equivalence. Xu et al.’s investigation [12] was conducted with an earlier wave of data collection from the study that was the source of data for the present investigation, The Adolescent Brain Cognitive Development (ABCD) Study (see Sample and Procedures). The authors investigated measurement non-equivalence with respect to sex and race/ethnicity, including White youth, using a standard approach, and found strong measurement equivalence (i.e., scalar; see Data Analysis) across sex and across race/ethnicity. However, notable gaps remain with respect to administration of this measure to youth. First, Xu et al. examined this measure with participants who were approximately 10 to 11 years of age. As perceptions of racial discrimination change with age [13], patterns of response to a racial discrimination measure cannot be assumed to remain constant. Second, the authors did not consider intersectional identity (i.e., assessing the intersection of sex and race/ethnicity), despite evidence that the direction of gender differences in reports of discrimination varies by racial/ethnic group [2, 5]. That is, girls from some racial/ethnic groups may report experiencing racial discrimination to a greater degree than boys, whereas girls from other racial/ethnic groups may report experiencing discrimination to a lesser degree. It is critical to ensure that valid comparisons can be made within as well as across gender and racial/ethnic groups. Third, Xu et al. did not investigate the possibility that measurement equivalence findings may differ when White youth, who are not the target population for racial discrimination measures and were not included in the development of the PDS, are included in the analysis.

To provide a broader context for the current study, we review findings from the application of measurement equivalence methods to other measures of racial or ethnic discrimination as well. Measurement equivalence has been examined in the Daily Life Experiences Scale [14] and Brief Perceived Ethnic Discrimination Scale [15] in addition to the widely used Everyday Discrimination Scale [16] in adults. Among these studies, measurement equivalence with respect to gender was examined - and observed - for the Daily Life Experiences Scale in a sample of African American college students [17]. Measurement equivalence with respect to race/ethnicity was assessed in the Brief Perceived Ethnic Discrimination Scale [18] and found across American Indian, African American, Latinx, East Asian, and South Asian adults. However, lack of measurement equivalence with respect to race/ethnicity has been observed in several studies examining the Everyday Discrimination Scale [19–21].

Far fewer studies have assessed measurement equivalence in racial discrimination measures administered to youth. Two such studies, examining the Perceived Racism for Youth and Children scale [22] and the Online Racial Discrimination subscales from the Online Victimization Scale [23] observed differences in intercepts across gender ([24] and [25], respectively). Notably, Hoang et al. [25] also assessed for measurement equivalence with respect to race/ethnicity on the Online Victimization Scale and found equivalence across Black and Latinx youth (the only two groups included). The lack of consistent evidence for measurement equivalence in other discrimination measures raises questions about the validity of reported gender or race/ethnicity differences in racial discrimination for any measure that has not undergone measurement equivalence testing. Notably, investigations drawing on ABCD Study data (the source for the current investigation) have used PDS scores without assessing and adjusting for measurement non-equivalence, both including White youth. One did not test for differences by race/ethnicity [26], but another examined trajectories of perceived discrimination over time and found consistently higher endorsement of perceived racial discrimination by Black relative to White youth [27].

### Aims of the Current Study

The aims of the current study were to assess measurement equivalence of the PDS with respect to sex (as a proxy for gender), race/ethnicity, and intersectional identity (sex by race/ethnicity), among middle-school-aged Black, Latinx, and White youth and to generate PDS scores adjusted for measurement non-equivalence. Additionally, to quantify the impact of measurement non-equivalence on group comparisons, we calculated scores both prior to and after adjusting for measurement non-equivalence and contrasted the pre- and post-adjustment group comparisons.

The validity of the label racial or ethnic discrimination for (non-Latinx) White youth’s experiences is inconsistent with the systemic inequality rooted in historic oppression and membership in a minority group, core components of discrimination [28]. However, racial discrimination measures are regularly administered to White participants and their scores are compared to those of participants who identify with racial/ethnic minority groups. Thus, regardless of whether we consider such comparisons to be valid, as White study participants are being administered racial discrimination scales and data from White respondents are being included in racial discrimination studies, it is important to understand how these measures perform in White youth, in addition and relative to youth of color. We therefore included data from White youth in the present investigation. Given the inconsistency of findings in the literature regarding measurement equivalence with respect to gender and race/ethnicity for other racial discrimination measures, we hypothesized only that we would identify measurement non-equivalence and did not formulate more specific hypotheses.

## Methods

### Sample and Procedures

The Adolescent Brain Cognitive Development (ABCD) Study is an ongoing longitudinal multi-site study of health and cognitive development of adolescents in the U.S. [https://abcdstudy.org]. Details of study design and ascertainment can be found in prior publications [29]. Briefly, from 2016 to 2018, 21 sites recruited 9- and 10-year-old youth and their primary caregiver (hereafter referred to as parent), drawing on enrollment data from the National Center for Education Statistics and data from the U.S. Census Bureau’s American Community Survey to derive ascertainment targets, applying probability sampling to target schools within catchment areas. A centralized Institutional Review Board approved study protocols. Written informed consent from a parent and assent from the child were obtained at the time of enrollment.

Surveys were administered to the child and parent in person (with the exception of online survey administration during the peak of the COVID-19 pandemic). A wide range of mental and physical health information and psychosocial factors were assessed. Data for the present study were drawn from Follow-up Year 2, release 5 (mean age = 12.03; SD = 0.10), the most recent publicly available wave of data collection in which the PDS was administered. We used data collected from the racial/ethnic groups with the highest representation in the sample: Black, Latinx, and White. This was because of the power limitations for detecting race/ethnicity differences among racial/ethnic groups with substantially lower representation in the sample (e.g., Asian, Native American), particularly when considering the intersection of sex by race/ethnicity. Race/ethnicity was categorized by the ABCD Study using parent reports of youth race (for the current study, race categories were Black/African American and White) and youth ethnicity (Hispanic/Latinx). Under the ABCD categorization scheme, all youth identified as Latinx/Hispanic were categorized as Latinx, such that “White” refers to Non-Latinx White and “Black” as Non-Latinx Black. Sex assigned at birth was used as a proxy for gender, as appropriate categorization (e.g., distinguishing cisgender from transgender girls and boys) would result in several categories with too few individuals to conduct sufficiently powered analyses.

The analytic sample was composed of 9,360 youth: 47.53% female; 15.90% Black, 22.42% Latinx, and 61.68% White. Just over half of parents reported college or higher education level (54.39%); 27.89% reported a household income below $50,000, 42.94% $100,000 or higher.

### Measures

#### Perceived Discrimination Scale (PDS)

The PDS [11] consists of seven items. The first three use the stem question, “How often do the following people treat you unfairly or negatively because of your ethnic background?” PDS-1 refers to teachers, PDS-2 refers other adults outside of school, and PDS-3 refers to other students. PDS-4. “I feel that others behave in an unfair or negative way toward my ethnic group” is not specific to a given group of individuals. The remaining items (PDS-5, PDS-6, and PDS-7) refer to perceptions of the attitudes of Americans broadly. Reponses were on a 5-point Likert scale: (1) *Almost never* to (5) *Very often*. Coefficient omega for the full sample was 0.68 (within the minimally acceptable range [30]). Group-specific omega values ranged from 0.62 (White females) to 0.69 (Latinx females). (Frequencies for response options of each PDS item by sex, race/ethnicity, and the six groups [Black females, Black males, Latinx females, Latinx males, White females, and White males] are reported in Supplemental Tables 4–6.)

#### Coding of Sex and Race/Ethnicity

Parent-reported youth sex (female, male) and race/ethnicity (Black, Latinx, White) and conditional classifications across sex and race/ethnicity were included as unordered categorical covariates. They were represented with a set of five orthogonal contrast-coded predictors reflecting a Helmert coding scheme. (1) Sex was coded as a single contrast and race/ethnicity was represented by a pair of contrast codes: (2) Black and Latinx versus White and (3) Black versus Latinx. Two contrast codes representing the sex by race/ethnicity interaction were derived via products of the code for sex and each of the race/ethnicity codes: (4) sex by Black and Latinx versus White; and (5) sex by Black versus Latinx. (See Table 1 for contrast codes.)

### Analytic Plan

Analyses proceeded in three steps. Following recommendations of Widaman and Reise [31], the extent of measurement equivalence across the six discrete groups (Black females, Black males, Latinx females, Latinx males, White females, and White males) was first assessed. Unidimensional multiple-group Confirmatory Factor Analysis (CFA) was conducted in M*plus* version 8.10 [32], comparing three standard, incrementally constrained models of measurement equivalence (configural, metric, and scalar). Individual models were evaluated via three criteria: χ^2^ [33], the comparative fit index (CFI), and root mean square error of approximation (RMSEA; [34]). Nested comparisons were evaluated using scaled χ^2^ difference tests; 90% confidence intervals (CIs) for RMSEA, with non-overlapping CIs indicating significant decrements in model fit [35]; and ΔCFI, where values between a less restricted and more restricted model greater than 0.01 indicate significant decrements in model fit [36].

Given evidence from the multiple-group CFA of inadequate cross-group equivalence (see Supplementary Table 1), we proceeded to the second stage of analysis, conducting MNLFA [10] in R (version 4.2.2) and M*plus* (version 8.10) using the semi-automated MNLFA R package developed for scale scoring (version 1.1.2; [9, 37]). We followed the procedures described in detail in our prior studies (e.g. [38]), in which we adjusted for measurement non-equivalence of scales administered in the ABCD Study. The R package generated a series of Mplus input files, which were used collectively to determine the extent to which sex, race/ethnicity, and interactions of sex by race/ethnicity moderated parameters defining the structural (factor mean and variance) and/or measurement (item intercepts and factor loadings) models for the PDS.

Initial MNLFA models independently assessed the impact of covariates on the mean and variance of the unidimensional latent PDS factor, and on individual item intercepts and factor loadings while holding all others invariant. In subsequent models, all sets of orthogonal contrast coded covariates (e.g., Black and Latinx versus White and Black versus Latinx) remained paired, such that retention of any one due to significance resulted in retention of the other(s). All effects (and paired effects) of sex, race/ethnicity, and sex by race/ethnicity exceeding either a significance threshold of *p* <.10 within initial mean and variance impact models or a threshold of *p* ≤.05 in relation to factor loadings and item intercepts in the item-specific measurement equivalence models were carried forward for simultaneous testing in a conditional model. Following convergence of the conditional model, all covariates and paired effects of covariates significant at *p* <.05 on the mean and variance of the latent factor were included in a final model. Significant and paired covariate effects were subjected to sequential trimming via the adoption of a 5% false discovery rate (FDR; [39]), applied first to loadings and then to intercepts, to guard against Type 1 error inflation. The retained parameters comprised the final version of the model from which bias-adjusted factor scores were derived.

In the third analytic stage, PDS scores were generated using the original (unadjusted) item values, according to the ABCD scoring procedure of calculating the mean across items, and pairwise comparisons of mean scores were tested across sex, race/ethnicity, and intersectional identity. Parallel analyses were then conducted with the MNLFA-adjusted PDS factor score. The ABCD weighting variable that accounts for ascertainment strategy was applied in all comparisons. A 5% FDR was applied in this series of analyses.

## Results

A unidimensional multiple-group CFA (Stage 1) indicated inadequate equivalence of measurement across the six groups, suggesting the necessity for MNLFA implementation to identify and adjust for sources of measurement bias. (As stated in the Analytic Plan, results are reported in Supplemental Table 1). Note that a sensitivity analysis was conducted, excluding White participants, and the adjusted scores generated for Black and Latinx participants did not differ from those generated in analyses including White participants, so we report on results from models using the full sample. (Results from the analyses conducted exclusively with Black and Latinx youth are available upon request.)

### MNLFA: Identifying and Adjusting for Sources of Measurement Non-Equivalence (Stage 2)

Results from the initial and simultaneous models are reported in Supplemental Tables 2 and 3. Results from the final model are described here and reported in Table 1. We found no evidence of differences by sex, race/ethnicity, or the interaction between sex and race/ethnicity in factor means or variance in PDS scores. At the individual item level, for five of the seven items, there was no evidence of non-equivalence, i.e., they were fully invariant. The two items for which non-equivalence was observed queried unfair or negative treatment in school by teachers (PDS-1) and other students (PDS-3) using the stem question, “How often do the following people treat you unfairly or negatively because of your ethnic background?” For both items, significant differences by sex were observed in intercepts, reflecting greater endorsement of these two items by female compared to male youth at the same level of the PDS factor score (i.e., level of perceived discrimination).

### Group Comparisons in Mean PDS Scores Pre- and Post- MNLFA Adjustments (Stage 3)

Results of pairwise testing of group differences in original (unadjusted) and post-MNLFA-adjusted PDS scores by sex, race/ethnicity, and intersectional (sex by race/ethnicity) identity are reported in Table 2. Means of unadjusted scores are shown in the top row. MNLFA-adjusted scores are shown in the left column. For each comparison, values of Hedges’ *g* [40]; with corresponding thresholds of 0.2 = small, 0.5 = medium, 0.8 = large effect sizes) are reported above the diagonal for unadjusted scores and below the diagonal for MNLFA-adjusted scores. For ease of interpretation, the larger of the two effect sizes (unadjusted mean or adjusted mean) for a given comparison is printed in black and the smaller effect size is printed in gray. Toward our aim of demonstrating the impact of measurement non-equivalence on drawing valid group comparisons, we summarize pre- to post-adjustment changes in magnitude of group differences.

No sex differences in mean PDS scores were observed either across or within racial/ethnic groups using unadjusted scores and no sex differences emerged when means were compared using adjusted scores. Differences in PDS scores were observed across racial/ethnic groups using unadjusted scores, specifically, significantly higher scores among Black youth relative to Latinx and White youth, and among Latinx youth relative to White youth, and they remained when comparisons were made using adjusted scores. Notably, the effect size for mean comparisons of Black and White youth increased from 0.784 to 0.851; although only a slight increase, the categorization of the effect size changed from the medium to large range. Likewise, the effect size for mean comparisons of Latinx and White youth increased, from a small (0.403) to medium (0.515) effect size. Some within-sex racial/ethnic group differences observed using unadjusted scores, specifically, higher scores among Black relative to White females, Latinx relative to White females, and Black relative to White males, also increased in effect size when using adjusted scores. Effect sizes increased from small (0.397) to medium (0.519) for the Latinx to White females comparison and from medium (0.770) to large (0.893) for the Black to White females comparison. Additionally, a very modest increase in effect size for the comparison of Black to White males shifted the effect size from medium (0.799) to large (0.809).

## Discussion

The current investigation of measurement equivalence of the PDS in Black, Latinx, and White middle-school-aged youth revealed equivalence with respect to sex, race/ethnicity and intersectional identity for the factor score and five of the seven items. Measurement non-equivalence was observed exclusively with respect to sex for the item querying treatment by teachers and the item querying treatment by other students. Specifically, the intercept differed by sex, reflecting greater endorsement of these two items by female compared to male youth at the same level of the PDS factor score (i.e., level of perceived discrimination). These findings do not support our overall hypothesis that we would observe a substantial degree of measurement non-equivalence reflected in different indicators (i.e., factor mean, factor variance, item intercept, factor loading) and more specifically that we would observe measurement non-equivalence with respect to race/ethnicity. Counter to expectations, we found no evidence to suggest that White youth interpreted and/or responded to the measure differently than their Black and Latinx peers. However, adjusting the scores for the modest degree of measurement non-equivalence resulted in minor but meaningful increases in the estimated magnitude of difference in PDS scores between White youth and the two minoritized groups. In other words, minimizing the measurement bias revealed greater true distinctions between Black and White youth and between Latinx and White youth in perceived racial discrimination, as measured by the PDS.

### Limited Measurement Non-Equivalence By Sex and Measurement Equivalence By Race/Ethnicity

Our findings of measurement non-equivalence with respect to sex are consistent with two of the known measurement equivalence studies of racial discrimination measures in youth, which found differences across gender in item thresholds [24, 25]. As both PDS items, frequency of being treated unfairly or negatively because of [their] ethnic background by (1) teachers and (2) other students, are specific to the school setting, the possibility that girls experience discrimination in school more frequently or differently than boys merits consideration. However, studies exploring potential gender differences in experiences of racial discrimination in school have either found no difference by gender [41, 42] or a higher frequency of peer and classroom discrimination experiences among boys relative to girls [43, 44]. Thus, although it is important to identify and correct for this response bias, the relevant literature does not suggest a likely source. Qualitative methods could be informative for understanding this pattern of response and, importantly, for refining racial discrimination measures administered to youth.

The observed measurement equivalence with respect to race/ethnicity is consistent with findings for the PDS in an earlier wave of data collection [12]. It is also consistent with findings of measurement equivalence for the Perceived Racism for Youth and Children scale from a sample of Black, Latinx, American Indian or Alaska Native, Asian, and Multiracial youth [24] and for the Brief Perceived Ethnic Discrimination Scale from a sample of American Indian, African American, Latinx, East Asian, and South Asian adults [18]. However, it was somewhat surprising considering the findings from most measurement equivalence studies of racial discrimination scales that include White participants (Xu et al. [12] being an exception). Prior investigations have found measurement non-equivalence with respect to race/ethnicity [20, 21], as might be expected, given the questionable applicability of the construct of racial discrimination to White individuals, which could result in response patterns that are distinct from individuals who identify as a member of a minoritized racial/ethnic group. Both studies examined the Everyday Discrimination Scale, which is worded differently (querying attribution of unfair treatment to various social identities rather than exclusively to race/ethnicity) from the PDS. The wording of discrimination items is known to impact endorsement [45, 46]. The observed measurement equivalence with respect to race/ethnicity in the current sample, in combination with the lower mean PDS scores among White relative to Black and Latinx youth, suggests that the wording of the PDS items captures the construct of racial discrimination well for this population, i.e., middle-school aged Black, Latinx, and White youth. Critically, results of sensitivity analyses revealed that the inclusion of White participants in the analytic sample did not impact the detection of or adjustment for measurement non-equivalence for Black and Latinx youth.

### Minor But Meaningful Shifts in Magnitude of Estimated Group Differences

The pattern of results of group comparisons on PDS scores after adjustment for measurement non-equivalence did not differ from those based on original (unadjusted) scores with respect to direction (e.g., higher for Black versus White youth). Likewise, no previously undetected differences emerged, and no previously observed differences were reduced to non-significance after adjusting for measurement non-equivalence. However, for numerous comparisons, there were minor increases (and no decreases) in the magnitude of effect sizes for group differences on PDS scores after adjustment for measurement non-equivalence. Interestingly, even though we found measurement equivalence with respect to race/ethnicity, the adjustments made to account for measurement non-equivalence with respect to sex impacted group differences by race/ethnicity, e.g., for Latinx versus White girls. Importantly, despite being only minor increases, they shifted the effect size from the small to medium range for differences between Latinx and White youth and from the medium to large range for differences between Black and White youth. As findings are often described in terms of small, medium, or large effects when summarizing results (e.g., citing prior literature), these modest changes in effect size result in a meaningful change in how group differences are characterized. Notably, adjustments for measurement non-equivalence revealed greater group differences in endorsement of discrimination experiences, which had been partially masked by measurement bias.

### Limitations

Certain limitations should be kept in mind when interpreting findings from the current study. First, we examined measurement equivalence with respect to sex and race/ethnicity in middle-school-aged Black, Latinx, and White youth. Inferences cannot be made about other age groups or racial/ethnic groups not included in the analyses. Relatedly, our categorization of race/ethnicity was constrained by the definitions adopted by the ABCD Study. Although consistency with the ABCD categorization scheme is beneficial with respect to the application of our findings to subsequent research conducted with this widely used public dataset, it limits the generalizability to samples that define race/ethnicity differently from the ABCD Study. In addition, due to limitations of the data for assessing measurement equivalence with respect to gender identity, we used sex as a proxy for gender. Moreover, limitations related to the psychometric properties of the PDS should be considered. In the current sample, the coefficient omega for the full sample was 0.68, with somewhat lower values for certain subgroups. Critiques of the PDS more generally include the possibility that a bifactor model is a better fit and that the skewness of responses may be indicative of an inappropriate response scale for pre-adolescent youth [12], suggesting the potential value of more extensive psychometric testing that would guide modification of the PDS based on developmental stage.

### Future Directions

As conceptualizations of racial/ethnic identity and discrimination experiences evolve with age, it is critical to assess and adjust for measurement equivalence on the PDS at older ages, which will be possible in the ABCD Study, as data collection will continue through early adulthood. The longitudinal nature of the ABCD Study will further allow for the assessment of measurement equivalence over the pre- to late adolescent years, an essential step in determining the extent to which the PDS is developmentally appropriate for capturing the construct of perceived racial discrimination across these developmental periods. There are of course much broader future directions in addition to those that can be pursued with data from the ABCD Study. Our findings support the value of considering and, where possible, adjusting for measurement non-equivalence with respect to gender (or sex) and race/ethnicity as a standard practice for any measure. As illustrated in our study by the finding of measurement equivalence with respect to race/ethnicity in a scale assessing racial discrimination in a sample that included White individuals, the nature (or absence) of possible measurement bias is not consistently intuitive. Race/ethnicity and sex are also by no means the only characteristics that can impact interpretations of and responses to items in various measures. Sexual orientation, age, socioeconomic status, and geographic region are just some of the other possible characteristics that should be considered in efforts to reduce measurement bias, to ensure that the experiences of individuals from all relevant backgrounds are accurately represented when measuring a given construct.

## Supplementary Material

Sartor supp

**Supplementary Information** The online version contains supplementary material available at https://doi.org/10.1007/s40615-026-02938-8.

## Figures and Tables

**Table 1:** Parameter estimates from the final moderated nonlinear factor analysis model

Reference Parameter	Sex Estimate (SE)	Race/Ethnicity 1 Estimate (SE)	Race/Ethnicity 2 Estimate (SE)	Sex × Race/Ethnicity 1 Estimate (SE)	Sex × Race/Ethnicity 2 Estimate (SE)
*Mean*	--	--	--	--	--
*Variance*	--	--	--	--	--
PDS-1. How often do the following people treat you unfairly or negatively because of your ethnic background?: **Teachers**					
*Intercept*	−0.21 (0.05)***	--	--	--	--
*Loading*	--	--	--	--	--
PDS-2. How often do the following people treat you unfairly or negatively because of your ethnic background? **Other adults outside of school**					
*Intercept*	--	--	--	--	--
*Loading*	--	--	--	--	--
PDS-3. How often do the following people treat you unfairly or negatively because of your ethnic background? **Other students**					
*Intercept*	−0.15 (0.03)***	--	--	--	--
*Loading*	--	--	--	--	--
PDS-4. I feel that others behave in an unfair or negative way toward my ethnic group.					
*Intercept*	--	--	--	--	--
*Loading*	--	--	--	--	--
PDS-5. I feel that I am not wanted in American society					
*Intercept*	--	--	--	--	--
*Loading*	--	--	--	--	--
PDS-6. I don’t feel accepted by other Americans.					
*Intercept*	--	--	--	--	--
*Loading*	--	--	--	--	--
PDS-7. I feel that other Americans have something against me.					
*Intercept*	--	--	--	--	--
*Loading*	--	--	--	--	--

Note.

* *p* <.05,

** *p* <.01,

*** *p* <.001.

Parameter coding: Sex (Female = 1, Male = −1), Race/Ethnicity 1 (Black = −1/3, Latinx = −1/3, White = 2/3,), Race/Ethnicity 2 (Black = −1/2, Latinx = 1/2, White = 0), Sex × Race/Ethnicity 1 (Black Female = −1/3, Black Male = 1/3, Latinx Female = −1/3, Latinx Male = 1/3, White Female = 2/3, White Male = −2/3), Sex × Race/Ethnicity 2 (Black Female = −1/2, Black Male = 1/2, Latinx Female = 1/2, Latinx Male = −1/2, White Female = 0, White Male = 0)

**Table 2:** Group sizes and means for sum (unadjusted) scores and adjusted factor scores, with effect sizes (Hedges’ g) for group differences

		Sex		Race/Ethnicity			Intersectional Identity					
		F	M	B	L	W	BF	BM	LF	LM	WF	WM
	***Mean (SD)***	*1.14(0.33)*	*1.14(0.32)*	*1.31(0.48)*	*1.19(0.37)*	*1.08(0.21)*	*1.30(0.47)*	*1.31(0.49)*	*1.18(0.38)*	*1.19(0.35)*	*1.08(22)*	*1.08(0.21)*
F	−*0.00(68)*	***n* = 4,449**	ns									
M	*0.00(66)*	ns	***n* = 4,911**									
B	*0.36(81)*			***n* = 1,488**	0.289	0.784						
L	*0.16(76)*			0.253	***n* = 2,099**	0.403						
W	−*0.15(53)*			0.851	0.515	***n* = 5,773**						
BF	*0.37(82)*						***n* = 747**	ns	0.276	0.278	0.770	0.772
BM	*0.34(80)*						ns	***n* = 741**	0.300	0.303	0.795	0.799
LF	*0.15(77)*						0.282	0.244	***n* = 1007**	ns	0.397	0.382
LM	*0.17(76)*						0.263	0.225	ns	***n* = 1,092**	0.426	0.409
WF	−*0.16(52)*						0.893	0.848	0.519	0.549	***n* = 2,695**	ns
WM	−*0.14(53)*						0.856	0.809	0.481	0.512	ns	***n* = 3,078**

*Note*. Gray shaded text boxes on the diagonal depict group-specific sample sizes, with corresponding means and standard deviations italicized under group labels atop the upper diagonal for PDS sum scores, and beside the left adjacent group labels below the diagonal for MNLFA-adjusted PDS factor scores; off-diagonal coefficients within the three boxes reflect Hedges’ g for significant group differences in PDS sum scores (above diagonal) and adjusted factor scores (below diagonal) given a False Discovery Rate of 5%; regarding absolute differences in effects derived from the two scoring methods, the larger effects are printed in black, with smaller effects in gray typeface; F=Female, M=Male, B=Black, L=Latinx, W=White, BF=Black Female, BM=Black Male, LF=Latinx Female; LM=Latinx Male, WF=White Female, WM=White Male, ns=nonsignificant
