# Supplementary material for: Measurement Equivalence Across Sex, Race/Ethnicity, and Intersectional Identity for the Perceived Discrimination Scale Among Middle-School-Aged Black, Latinx, and White Youth: Findings From the Adolescent Brain Cognitive Development Study
Source: J Racial Ethn Health Disparities. Author manuscript; Available in PMC 2026 May 27. (PMC13213479; doi:10.1007/s40615-026-02938-8)
Supplement: Sartor supp [file NIHMS2177791-supplement-Sartor_supp.docx]

Supplemental Tables

Measurement equivalence across sex, race/ethnicity, and intersectional identity for the Perceived Discrimination Scale among middle-school-aged Black, Latinx, and White youth: Findings from findings the Adolescent Brain Cognitive Development Study

Journal of Racial and Ethnic Health Disparities

Carolyn E. Sartor, Margret Z. Powell, Nicole Kennelly, Tammy Chung, & Shawn J. Latendresse

Corresponding author: Carolyn E. Sartor, Institute for Health, Health Care Policy and Aging Research, Rutgers University, 112 Paterson Street, New Brunswick, NJ 08901, csartor@ifh.rutgers.edu

Supplemental Table 1. Measurement equivalence testing via multiple-group CFA

|  |  |  |  |  |  |  |  |  |  |  |  |  |
| --- | --- | --- | --- | --- | --- | --- | --- | --- | --- | --- | --- | --- |
| ***Model Fit*** | | | | | |  | ***Model Comparisons*** | | | | | |
| **Model** | ***𝛘* ^2^** | **df** | ***p*-value** | **RMSEA (90%CI)** | **CFI** |  | **Comparison** | 𝚫***𝛘* ^2^** ^a^ | **df** | ***p*-value** | **CI Overlap** | 𝚫**CFI** |
| Configural | 1275.01 | 84 | 0.000 | .095 (.091, .100) | .530 |  | na |  |  |  |  |  |
| Metric | 1131.49 | 114 | 0.000 | .076 (.072, .080) | .598 |  | configural | 43.894 | 30 | 0.049 | no | .068 |
| Scalar | 1558.01 | 149 | 0.000 | .078 (.074, .081) | .444 |  | metric | 631.33 | 35 | 0.000 | no | -.154 |
|  |  |  |  |  |  |  |  |  |  |  |  |  |
|  |  |  |  |  |  |  |  |  |  |  |  |  |

*Note*. Assessing the extent of measurement equivalence (configural, metric, scalar) in Perceived Discrimination Scale scores across six intersectional identities was achieved via the following three tests for the comparison of nested models: the Satorra-Bentler scaled chi-square difference test (Satorra & Bentler, 2001), overlap among the 90% confidence intervals for RMSEA (Wang & Russell, 2005), and absolute change in CFI (Cheung & Rensvold, 2002). CFA = confirmatory factor analysis; RMSEA = root mean square error of approximation; CI = confidence interval; CFI = comparative fit index.

^a^ Changes in model chi-square estimates derived via robust maximum likelihood (MLR in M*plus*; Muthén & Muthén, 2017) requires use of the Satorra-Bentler scaled chi-square difference test.

Supplemental Table 2. Parameter estimates from initial moderated nonlinear factor analysis model

| **Reference**  ***Parameter*** | ***Sex***  *Estimate (SE)* | ***Race/Ethnicity 1***  *Estimate (SE)* | ***Race/Ethnicity 2***  *Estimate (SE)* | ***Sex x***  ***Race/Ethnicity 1***  *Estimate (SE)* | ***Sex***  ***x Race/Ethnicity 2***  *Estimate (SE)* |
| --- | --- | --- | --- | --- | --- |
| *Mean* | -0.02 (0.02) | -0.81 (0.03)*** | -0.28 (0.03)*** | -0.05 (0.03) | -0.04 (0.04) |
| *Variance* | 0.08 (0.04)* | 0.14 (0.07)* | 0.26 (0.10)** | 0.01 (0.07) | 0.04 (0.10) |
| PDS-1. How often do the following people treat you unfairly or negatively because of your ethnic background? | | | | | |
| *Intercept* | -0.27 (0.10)** | -1.54 (0.22)*** | -1.12 (0.23)*** | 0.00 (0.22) | -0.25 (0.23) |
| *Loading* | 0.05 (0.08) | 0.85 (0.17)*** | 0.31 (0.17) | 0.00 (0.17) | 0.31 (0.17) |
| PDS-2. How often do the following people treat you unfairly or negatively because of your ethnic background? | | | | | |
| *Intercept* | -.20 (0.11) | -0.85 (0.21)*** | -0.40 (0.27) | -0.10 (0.21) | -0.26 (0.27) |
| *Loading* | -0.04 (0.08) | 0.60 (0.17)*** | -0.02 (0.20) | -0.07 (0.17) | 0.18 (0.20) |
| PDS-3. How often do the following people treat you unfairly or negatively because of your ethnic background? | | | | | |
| *Intercept* | -0.19 (0.06)** | -0.55 (0.12)*** | -0.28 (0.17) | 0.18 (0.12) | -0.28 (0.17) |
| *Loading* | 0.03 (0.06) | 0.67 (0.12)*** | 0.10 (0.14) | -0.08 (0.12) | 0.17 (0.14) |
| PDS-4. I feel that others behave in an unfair or negative way toward my ethnic group. | | | | | |
| *Intercept* | 0.09 (0.06) | -1.12 (0.12)*** | -0.44 (0.14)** | -0.11 (0.12) | 0.07 (0.14) |
| *Loading* | -0.06 (0.05) | 0.69 (0.12)*** | 0.11 (0.12) | 0.08 (0.12) | 0.00 (0.12) |
| PDS-5. I feel that I am not wanted in American society | | | | | |
| *Intercept* | 0.12 (0.22) | -2.32 (0.52)*** | -0.92 (0.49) | -0.08 (0.52) | 0.24 (0.49) |
| *Loading* | -0.02 (0.15) | 0.97 (0.34)** | 0.59 (0.31) | -0.08 (0.34) | -0.31 (0.31) |
| PDS-6. I don't feel accepted by other Americans. | | | | | |
| *Intercept* | 0.18 (0.26) | -0.48 (0.52) | 1.86 (0.70)** | -0.99 (0.52) | -0.70 (0.68) |
| *Loading* | 0.04 (0.16) | 0.00 (0.33) | -0.83 (0.42)* | 0.65 (0.33) | 0.39 (0.41) |
| PDS-7. I feel that other Americans have something against me. | | | | | |
| *Intercept* | 0.39 (0.20) | -1.81 (0.44)*** | 0.25 (0.47) | -0.02 (0.44) | 0.32 (0.47) |
| *Loading* | -0.14 (0.13) | 0.90 (0.30)** | -0.06 (0.31) | 0.13 (0.30) | -0.20 (0.31) |

*Note*. **p* < .05, ***p* < .01, ****p* < .001. Parameter coding: Sex (Female = 1, Male = -1), Race/Ethnicity 1 (Black = -1/3, Latinx = -1/3, White = 2/3,), Race/Ethnicity 2 (Black = -1/2, Latinx = 1/2, White = 0), Sex x Race/Ethnicity 1 (Black Female = -1/3, Black Male = 1/3, Latinx Female = -1/3, Latinx Male = 1/3, White Female = 2/3, White Male = -2/3), Sex x Race/Ethnicity 2 (Black Female = -1/2, Black Male = 1/2, Latinx Female = 1/2, Latinx Male = -1/2, White Female = 0, White Male = 0).

Supplemental Table 3. Parameter estimates from simultaneous moderated nonlinear factor analysis model

| **Reference**  ***Parameter*** | ***Sex***  *Estimate (SE)* | ***Race/Ethnicity 1***  *Estimate (SE)* | ***Race/Ethnicity 2***  *Estimate (SE)* | ***Sex x***  ***Race/Ethnicity 1***  *Estimate (SE)* | ***Sex***  ***x Race/Ethnicity 2***  *Estimate (SE)* |
| --- | --- | --- | --- | --- | --- |
| *Mean* | 0.00 (0.02) | -0.51 (0.75) | -0.25 (0.57) | -0.04 (0.03) | -0.05 (0.04) |
| *Variance* | 0.03 (0.03) | -0.05 (0.48) | 0.03 (0.34) | -- | -- |
| PDS-1. How often do the following people treat you unfairly or negatively because of your ethnic background? | | | | | |
| *Intercept* | -0.24 (0.05)*** | -1.30 (1.48) | -0.93 (0.83) | -- | -- |
| *Loading* | -- | 0.67 (0.47) | 0.24 (0.29) | -- | -- |
| PDS-2. How often do the following people treat you unfairly or negatively because of your ethnic background? | | | | | |
| *Intercept* | -- | -0.67 (1.56) | -0.35 (0.95) | -- | -- |
| *Loading* | -- | 0.47 (0.50) | 0.01 (0.34) | -- | -- |
| PDS-3. How often do the following people treat you unfairly or negatively because of your ethnic background? | | | | | |
| *Intercept* | -0.16 (0.04)*** | -0.33 (1.48) | -0.16 (0.90) | -- | -- |
| *Loading* | -- | 0.45 (0.48) | 0.07 (0.31) | -- | -- |
| PDS-4. I feel that others behave in an unfair or negative way toward my ethnic group. | | | | | |
| *Intercept* | -- | -0.76 (1.36) | -0.32 (0.83) | -- | -- |
| *Loading* | -- | 0.38 (0.42) | 0.08 (0.28) | -- | -- |
| PDS-5. I feel that I am not wanted in American society | | | | | |
| *Intercept* | -- | -1.60 (2.86) | -0.56 (1.87) | -- | -- |
| *Loading* | -- | 0.43 (0.79) | 0.43 (0.60) | -- | -- |
| PDS-6. I don't feel accepted by other Americans. | | | | | |
| *Intercept* | -- | -0.26 (2.79) | 1.31 (2.13) | -- | -- |
| *Loading* | -- | -0.28 (0.80) | -0.52 (0.68) | -- | -- |
| PDS-7. I feel that other Americans have something against me. | | | | | |
| *Intercept* | -- | -1.14 (2.82) | 0.29 (1.88) | -- | -- |
| *Loading* | -- | 0.34 (0.80) | -0.05 (0.59) | -- | -- |

*Note*. **p* < .05, ***p* < .01, ****p* < .001. Parameter coding: Sex (Female = 1, Male = -1), Race/Ethnicity 1 (Black = -1/3, Latinx = -1/3, White = 2/3,), Race/Ethnicity 2 (Black = -1/2, Latinx = 1/2, White = 0), Sex x Race/Ethnicity 1 (Black Female = -1/3, Black Male = 1/3, Latinx Female = -1/3, Latinx Male = 1/3, White Female = 2/3, White Male = -2/3), Sex x Race/Ethnicity 2 (Black Female = -1/2, Black Male = 1/2, Latinx Female = 1/2, Latinx Male = -1/2, White Female = 0, White Male = 0).

Supplemental Table 4. Frequencies of Perceived Discrimination Scale (PDS) item responses by sex

| **Response** | ***PDS-1***  *n (%)* | ***PDS-2***  *n (%)* | | ***PDS-3***  *n (%)* | ***PDS-4***  *n (%)* | ***PDS-5***  *n (%)* | | ***PDS-6***  *n (%)* | ***PDS-7***  *n (%)* |
| --- | --- | --- | --- | --- | --- | --- | --- | --- | --- |
| **Female** | | |  | | | |  | | |
| *1- Almost never* | 4175 (93.8) | 4151 (93.3) | | 3731 (83.9) | 3658 (82.2) | 4151 (93.3) | | 4139 (93.0) | 4080 (91.7) |
| *2- Rarely* | 123 (2.8) | 151 (3.4) | | 416 (9.4) | 358 (8.0) | 133 (3.0) | | 154 (3.5) | 187 (4.2) |
| *3 - Sometimes* | 55 (1.2) | 54 (1.2) | | 168 (3.8) | 203 (4.6) | 91 (2.0) | | 78 (1.8) | 102 (2.3) |
| *4 - Often* | 17 (0.4) | 11 (0.5) | | 45 (1.0) | 67 (1.5) | 15 (0.3) | | 17 (0.4) | 21 (0.5) |
| *5 - Very often* | <10 * | <10 * | | 26 (0.6) | 33 (0.7) | 19 (0.4) | | 16 (0.4) | 18 (0.4) |
| *777 - Don’t know* | 66 (1.5) | 77 (1.7) | | 58 (1.3) | 128 (2.9) | 40 (0.9) | | 45 (1.0) | 40 (0.9) |
| *Did not answer* | <10 * | <10 * | | <10 * | <10 * | <10 * | | <10 * | <10 * |
| **Male** |  |  | |  |  |  | |  |  |
| *1- Almost never* | 4508 (91.8) | 4457 (90.8) | | 3985 (81.1) | 4061 (82.7) | 4635 (94.4) | | 4643 (94.5) | 4584 (93.3) |
| *2- Rarely* | 175 (3.6) | 218 (4.4) | | 546 (11.1) | 426 (8.7) | 138 (2.8) | | 142 (2.9) | 180 (3.7) |
| *3 - Sometimes* | 77 (1.6) | 78 (1.6) | | 197 (4.0) | 169 (3.4) | 51 (1.0) | | 44 (0.9) | 61 (1.2) |
| *4 - Often* | 35 (0.7) | 25 (0.5) | | 67 (1.4) | 85 (1.7) | 13 (0.3) | | 16 (0.3) | 21 (0.4) |
| *5 - Very often* | 27 (0.5) | 13 (0.3) | | 47 (1.0) | 35 (0.7) | 13 (0.3) | | 21 (0.4) | 13 (0.3) |
| *777 - Don’t know* | 84 (1.7) | 113 (2.3) | | 63 (1.3) | 131 (2.7) | 56 (1.1) | | 41 (0.8) | 47 (1.0) |
| *Did not answer* | <10 * | <10 * | | <10 * | <10 * | <10 * | | <10 * | <10 * |

*Note.* * Cell counts below 10 reported as <10. ABCD mean scoring allowed for up to 3 of the 7 items to be missing; hence the presence of “Don’t know” responses and non-responses.

Supplemental Table 5. Frequencies of Perceived Discrimination Scale (PDS) item responses by race/ethnicity

| **Response** | ***PDS-1***  *n (%)* | ***PDS-2***  *n (%)* | | ***PDS-3***  *n (%)* | ***PDS-4***  *n (%)* | ***PDS-5***  *n (%)* | | ***PDS-6***  *n (%)* | ***PDS-7***  *n (%)* |
| --- | --- | --- | --- | --- | --- | --- | --- | --- | --- |
| **Black** | | |  | | | |  | | |
| *1- Almost never* | 1223 (82.2) | 1247 (83.8) | | 1092 (73.4) | 1014 (68.1) | 1274 (85.6) | | 1310 (88.0) | 1254 (84.3) |
| *2- Rarely* | 88 (5.9) | 106 (7.1) | | 180 (12.1) | 178 (12.0) | 89 (6.0) | | 70 (4.7) | 117 (7.9) |
| *3 - Sometimes* | 71 (4.8) | 48 (3.2) | | 110 (7.4) | 128 (8.6) | 60 (4.0) | | 50 (3.4) | 55 (3.7) |
| *4 - Often* | 24 (1.6) | 19 (1.3) | | 40 (2.7) | 62 (4.2) | 18 (1.2) | | 13 (0.9) | 17 (1.1) |
| *5 - Very often* | 21 (1.4) | <10 * | | 31 (2.1) | 38 (2.6) | 14 (0.9) | | 16 (1.1) | 17 (1.1) |
| *777 - Don’t know* | 57 (3.8) | 58 (5.8) | | 32 (2.2) | 67 (4.5) | 32 (2.2) | | 28 (1.9) | 26 (1.7) |
| *Did not answer* | <10 * | <10 * | | <10 * | <10 * | <10 * | | <10 * | <10 * |
| **Latinx** |  |  | |  |  |  | |  |  |
| *1- Almost never* | 1916 (91.3) | 1897 (90.4) | | 1651 (78.7) | 1613 (76.8) | 1883 (89.7) | | 1866 (88.9) | 1846 (87.9) |
| *2- Rarely* | 86 (4.1) | 88 (4.2) | | 266 (12.7) | 228 (10.9) | 102 (4.9) | | 128 (6.1) | 124 (5.9) |
| *3 - Sometimes* | 26 (1.2) | 41 (2.0) | | 93 (4.4) | 111 (5.3) | 61 (2.9) | | 44 (2.1) | 69 (3.3) |
| *4 - Often* | 16 (0.8) | 10 (0.5) | | 28 (1.3) | 43 (2.0) | <10 * | | 16 (0.8) | 10 (0.5) |
| *5 - Very often* | <10 * | <10 * | | 18 (0.9) | 14 (0.7) | 10 (0.5) | | 12 (1.6) | <10 * |
| *777 - Don’t know* | 46 (2.2) | 56 (2.7) | | 41 (2.0) | 88 (4.2) | 31 (1.5) | | 30 (1.4) | 38 (1.8) |
| *Did not answer* | <10 * | <10 * | | <10 * | <10 * | <10 * | | <10 * | <10 * |
| **White** |  |  | |  |  |  | |  |  |
| *1- Almost never* | 5544 (96.0) | 5464 (94.6) | | 4973 (86.1) | 5092 (88.2) | 5629 (97.5) | | 5606 (97.1) | 5564 (96.4) |
| *2- Rarely* | 124 (2.1) | 175 (3.0) | | 516 (8.9) | 378 (6.5) | 80 (1.4) | | 98 (1.7) | 126 (2.2) |
| *3 - Sometimes* | 35 (0.6) | 43 (0.7) | | 162 (2.8) | 133 (2.3) | 21 (0.4) | | 28 (0.5) | 39 (0.7) |
| *4 - Often* | 12 (0.2) | <10 * | | 44 (0.8) | 47 (0.8) | <10 * | | <10 * | 15 (0.3) |
| *5 - Very often* | <10 * | <10 * | | 24 (0.4) | 16 (0.3) | <10 * | | <10 * | <10 * |
| *777 - Don’t know* | 47 (0.8) | 76 (1.3) | | 48 (0.8) | 104 (1.8) | 33 (0.6) | | 28 (0.5) | 23 (0.4) |
| *Did not answer* | <10 * | <10 * | | <10 * | <10 * | <10 * | | <10 * | <10 * |

*Note.* * Cell counts below 10 reported as <10. ABCD mean scoring allowed for up to 3 of the 7 items to be missing; hence the presence of “Don’t know” responses and non-responses.

Supplemental Table 6. Frequencies of Perceived Discrimination Scale (PDS) item responses by intersectional identity (sex by race/ethnicity)

| **Response** | ***PDS-1***  *n (%)* | ***PDS-2***  *n (%)* | ***PDS-3***  *n (%)* | ***PDS-4***  *n (%)* | ***PDS-5***  *n (%)* | ***PDS-6***  *n (%)* | ***PDS-7***  *n (%)* |
| --- | --- | --- | --- | --- | --- | --- | --- |
| **Black Female** |  |  |  |  |  |  |  |
| *1- Almost never* | 636 (85.1) | 637 (85.3) | 562 (75.2) | 510 (68.3) | 624 (83.5) | 643 (86.1) | 620 (83.0) |
| *2- Rarely* | 33 (4.4) | 50 (6.7) | 78 (10.4) | 81 (10.8) | 44 (5.9) | 43 (5.8) | 60 (8.0) |
| *3 - Sometimes* | 35 (4.7) | 23 (3.1) | 57 (7.6) | 69 (9.2) | 42 (5.6) | 34 (4.6) | 36 (4.8) |
| *4 - Often* | <10 * | <10 * | 15 (2.0) | 30 (4.0) | 12 (1.6) | <10 * | <10 * |
| *5 - Very often* | <10 * | <10 * | 12 (1.6) | 17 (2.3) | <10 * | <10 * | <10 * |
| *777 - Don’t know* | 28 (3.7) | 29 (3.9) | 21 (2.8) | 40 (5.4) | 17 (2.3) | 15 (2.0) | 13 (1.7) |
| *Did not answer* | <10 * | <10 * | <10 * | <10 * | <10 * | <10 * | <10 * |
| **Black Male** |  |  |  |  |  |  |  |
| *1- Almost never* | 587 (79.2) | 610 (85.3) | 530 (71.5) | 504 (68.0) | 650 (87.7) | 667 (90.0) | 634 (85.6) |
| *2- Rarely* | 55 (7.4) | 56 (6.7) | 102 (13.8) | 97 (13.1) | 45 (6.1) | 27 (3.6) | 57 (7.7) |
| *3 - Sometimes* | 36 (4.9) | 25 (3.1) | 53 (7.2) | 59 (8.0) | 18 (2.4) | 16 (2.2) | 19 (2.6) |
| *4 - Often* | 16 (2.2) | 13 (0.8) | 25 (3.4) | 32 (4.3) | <10 * | <10 * | <10 * |
| *5 - Very often* | 16 (2.2) | <10 * | 19 (2.6) | 21 (2.8) | <10 * | 10 (1.3) | <10 * |
| *777 - Don’t know* | 29 (3.9) | 29 (3.9) | 11 (1.5) | 27 (3.6) | 15 (2.0) | 13 (1.8) | 13 (1.8) |
| *Did not answer* | <10 * | <10 * | <10 * | <10 * | <10 * | <10 * | <10 * |
| **Latinx Female** |  |  |  |  |  |  |  |
| *1- Almost never* | 930 (92.4) | 923 (91.7) | 822 (81.6) | 766 (76.1) | 899 (89.3) | 884 (87.8) | 876 (87.0) |
| *2- Rarely* | 36 (3.6) | 44 (4.4) | 114 (11.3) | 108 (10.7) | 48 (4.8) | 66 (6.6) | 59 (5.9) |
| *3 - Sometimes* | 11 (1.1) | 14 (1.4) | 37 (3.7) | 67 (6.7) | 39 (3.9) | 27 (2.7) | 45 (4.5) |
| *4 - Often* | <10 * | <10 * | <10 * | 18 (1.8) | <10 * | <10 * | <10 * |
| *5 - Very often* | <10 * | <10 * | <10 * | <10 * | <10 * | <10 * | <10 * |
| *777 - Don’t know* | 20 (2.0) | 21 (2.1) | 19 (1.9) | 39 (3.9) | 12 (1.2) | 17 (1.7) | 17 (1.7) |
| *Did not answer* | <10 * | <10 * | <10 * | <10 * | <10 * | <10 * | <10 * |

*Note.* * Cell counts below 10 reported as <10. ABCD mean scoring allowed for up to 3 of the 7 items to be missing; hence the presence of “Don’t know” responses and non-responses.

Supplemental Table 6. Frequencies of Perceived Discrimination Scale (PDS) item responses by intersectional identity (sex by race/ethnicity) continued

| **Response** | ***PDS-1***  *n (%)* | ***PDS-2***  *n (%)* | ***PDS-3***  *n (%)* | ***PDS-4***  *n (%)* | ***PDS-5***  *n (%)* | ***PDS-6***  *n (%)* | ***PDS-7***  *n (%)* |
| --- | --- | --- | --- | --- | --- | --- | --- |
| **Latinx Male** |  |  |  |  |  |  |  |
| *1- Almost never* | 986 (90.3) | 974 (89.2) | 829 (75.9) | 847 (77.6) | 984 (90.1) | 982 (89.9) | 970 (88.8) |
| *2- Rarely* | 50 (4.6) | 44 (4.0) | 152 (13.9) | 120 (11.0) | 54 (4.9) | 62 (5.7) | 65 (6.0) |
| *3 - Sometimes* | 15 (1.4) | 27 (2.5) | 56 (5.1) | 44 (4.0) | 22 (2.0) | 17 (1.6) | 24 (2.2) |
| *4 - Often* | <10 * | <10 * | 19 (1.7) | 25 (2.3) | 6 (0.5) | <10 * | <10 * |
| *5 - Very often* | <10 * | <10 * | 12 (1.1) | <10 * | <10 * | <10 * | <10 * |
| *777 - Don’t know* | 26 (2.4) | 35 (3.2) | 22 (2.0) | 49 (4.5) | 19 (1.7) | 13 (1.2) | 21 (1.9) |
| *Did not answer* | <10 * | <10 * | <10 * | <10 * | <10 * | <10 * | <10 * |
| **White Female** |  |  |  |  |  |  |  |
| *1- Almost never* | 2609 (96.8) | 2591 (96.1) | 2347 (87.1) | 2382 (88.4) | 2628 (97.5) | 2612 (96.9) | 2584 (95.9) |
| *2- Rarely* | 54 (2.0) | 57 (2.1) | 224 (8.3) | 169 (6.3) | 41 (1.5) | 45 (1.7) | 68 (2.5) |
| *3 - Sometimes* | 9 (0.3) | 17 (0.6) | 74 (2.7) | 67 (2.5) | 10 (0.4) | 17 (0.6) | 21 (0.8) |
| *4 - Often* | <10 * | <10 * | 21 (0.8) | 19 (0.7) | <10 * | <10 * | <10 * |
| *5 - Very often* | <10 * | <10 * | <10 * | <10 * | <10 * | <10 * | <10 * |
| *777 - Don’t know* | 18 (0.7) | 27 (1.0) | 18 (0.7) | 49 (1.8) | 11 (0.4) | 13 (0.5) | 10 (0.4) |
| *Did not answer* | <10 * | <10 * | <10 * | <10 * | <10 * | <10 * | <10 * |
| **White Male** |  |  |  |  |  |  |  |
| *1- Almost never* | 2935 (95.4) | 2873 (93.3) | 2626 (85.3) | 2710 (88.0) | 3001 (97.5) | 2994 (97.3) | 2980 (96.8) |
| *2- Rarely* | 70 (2.3) | 118 (3.8) | 292 (9.5) | 209 (6.8) | 39 (1.3) | 53 (1.7) | 58 (1.9) |
| *3 - Sometimes* | 26 (0.8) | 26 (0.8) | 88 (2.9) | 66 (2.1) | 11 (0.4) | 11 (0.4) | 18 (0.6) |
| *4 - Often* | 11 (0.4) | <10 * | 23 (0.7) | 28 (0.9) | <10 * | <10 * | <10 * |
| *5 - Very often* | <10 * | <10 * | 16 (0.5) | <10 * | <10 * | <10 * | <10 * |
| *777 - Don’t know* | 29 (0.9) | 49 (1.6) | 30 (1.0) | 55 (1.8) | 22 (0.7) | 15 (0.5) | 13 (0.4) |
| *Did not answer* | <10 * | <10 * | <10 * | <10 * | <10 * | <10 * | <10 * |

*Note.* * Cell counts below 10 reported as <10. ABCD mean scoring allowed for up to 3 of the 7 items to be missing; hence the presence of “Don’t know” responses and non-responses.

References

1. Cheung GW, Rensvold RB. Evaluating goodness-of-fit indexes for testing measurement invariance. Struct Equ Modeling. 2002;9:233-55. https://doi.org/10.1207/S15328007SEM0902_5

2. Muthén LK, Muthén BO. Mplus User’s Guide. 8th ed. Los Angeles, CA: Muthén & Muthén; 2017.

3. Satorra A, Bentler PM. A scaled difference chi-square test statistic for moment structure analysis. Psychometrika. 2001;66(4):507-14. https://doi.org/10.1007/BF02296192

4. Wang M, Russell SS. Measurement equivalence of the Job Descriptive Index across Chinese and American workers: Results from confirmatory factor analysis and item response theory. Educ Psychol Meas. 2005;65:709–32. https://doi.org/10.1177/0013164404272494
